# Targeting TRPV1 activity via high‐dose capsaicin in patients with sickle cell disease

**DOI:** 10.1002/jha2.528

**Published:** 2022-07-19

**Authors:** Alexander K. Glaros, Michael U. Callaghan, Wally R. Smith, Ahmar U. Zaidi

**Affiliations:** ^1^ Central Michigan University College of Medicine Mount Pleasant Michigan USA; ^2^ Division of Pediatric Hematology‐Oncology Children's Hospital of Michigan Detroit Michigan USA; ^3^ Division of General Internal Medicine Virginia Commonwealth University Richmond Virginia USA

**Keywords:** capsaicin, chronic pain, neuropathic pain, quantitative sensory testing, sickle cell disease

## Abstract

Evidence suggests neuropathic pain (NP) develops over time in sickle cell disease (SCD), contributing to a complex, difficult‐to‐treat phenotype, with management based on scant evidence. One characteristic of NP found is hyperalgesia caused by nervous system sensitization, but risk factors for this have not been identified within the SCD population, as exact mechanisms leading to its development are not well defined. The SPICE (Sickle cell Pain: Intervention with Capsaicin Exposure) trial was a pilot safety and feasibility trial of high‐dose (8%) topical capsaicin for patients with SCD and recurrent/chronic pain with neuropathic features, aimed at exploring capsaicin's utility as a mechanistic probe and adjunctive pain treatment for this population. Ten participants identifying “target” sites of pain with NP‐type qualities consented to treatment. The primary endpoint was safety/tolerability. The novel Localized Peripheral Hypersensitivity Relief score (LPHR) was developed to determine improvement in sensitivity attributable to TRPV1 neutralization. There were no severe treatment‐related adverse events. Higher baseline pain sensitivity at a given body site was associated with self‐reported history of more frequent localized vaso‐occlusive pain episodes at that site. There was a statistically significant improvement in the mean LPHR, evidencing TRPV1's importance to the development of hypersensitivity and a potential therapeutic benefit of capsaicin for SCD.

## 1 INTRODUCTION

Patients with sickle cell disease (SCD) experience pain far more often than is apparent from healthcare utilization, with variable clinical pain phenotypes [1, 2, 3]. Dampier et al. in their diagnostic criteria for chronic pain developed as part of The Analgesic, Anesthetic, and Addiction Clinical Trial Translations Innovations Opportunities and Networks‐American Pain Society Pain Taxonomy (AAPT) recently defined chronic SCD pain as ongoing pain present on most days in a 6‐month period, with either associated abnormal physical exam findings (e.g., weakness, tenderness) or imaging findings to suggest a contributory complication of SCD (e.g., avascular necrosis, skin ulcer) [2]. A subset of this, “chronic pain without contributory disease complications” has been established as the sum of variable contributions from nociceptive, inflammatory, and neuropathic pain (NP) types, providing an important framework for needed mechanistic and therapeutic investigation that have previously been lacking [2].

NP and neuropathy remain especially underinvestigated and undertreated causes of suffering among patients with SCD [4]. Patient‐reported outcome (PRO) measures indicate NP prevalence among SCD patients of 25%–40% [5, 6, 7, 8]. Additionally, studies utilizing quantitative sensory testing have found lower pain thresholds in patients with SCD, further evidencing the somatosensory sensitization that underlies NP [6, 9, 10]. There is surely some element of sensitization prior to the development of “screen‐positive” NP that contributes to worsening of pain phenotypes during the evolution from acute to chronic pain, but further research is needed regarding appropriate assessment tools for its identification.

A complete model for acute vaso‐occlusive pain progressing to the sensitization and chronic NP common in adolescents and adults remains elusive, but many of the involved maladaptive changes have been described [3, 11]. Human studies have demonstrated elevated substance P at baseline in patients with SCD, as well as aberrant pain processing pathways on functional magnetic resonance imaging and electroencephalograms [12, 13, 14, 15]. Murine investigations have demonstrated upregulation of the nociceptive ion channel transient receptor potential vanilloid‐1 (TRPV1), effects of immune cells on glial activation and neuroinflammation, and central sensitization with expansion of receptive fields at the level of the dorsal horn [16, 17, 18, 19, 20]. Much of the biological data are limited to murine models, but because of the availability of an FDA‐approved direct agonistic inhibitor of TRPV1, high‐dose (8%) topical capsaicin (Qutenza, Averitas Pharma), that receptor is well suited as a starting point for the back‐translation of these mechanisms into human models of SCD pain.

Repeated, intense activation of TRPV1‐expressing nociceptors results in upregulated TRPV1 signalling via decreased firing threshold and increased channel density in the affected and surrounding axons [16, 21, 22, 23]. This upregulation is seen in murine models of SCD, indicating its potentially important role in the transition from recurrent vaso‐occlusive pain to peripheral sensitization, and perhaps subsequently to central sensitization [17]. The 8% topical capsaicin, an excitotoxin causing reversible degeneration of sensitized, TRPV1‐expressing nociceptive neuronal axons has proven as efficacious as gabapentinoids in treating NP in certain disease states, but has not been studied in SCD [24, 25]. Knowing the mechanism of capsaicin in neutralizing TRPV1 upregulation, we designed a pilot investigation to primarily assess its safety in patients with SCD, as well as to explore its utility as a mechanistic probe in isolating the role of peripheral TRPV1 receptors in human SCD‐related pain toward future construction of a more detailed cause‐and‐effect model of chronic pain in SCD. We hypothesized that capsaicin would be well tolerated and would result in reduced pain sensitivity in treated areas, demonstrating its feasibility as a probe for future investigations.

## 2 METHODS

### 2.1 Study design

The SPICE pilot study (Sickle cell Pain: Intervention with Capsaicin Exposure; clinicaltrials.gov NCT03899246) was investigator initiated. It was approved by the Institutional Review Boards of Wayne State University and the Detroit Medical Center as an open‐label, single‐arm safety investigation of off‐label use of an FDA‐approved medication. Written informed consent was obtained from all participants ≥18 years old and from a legal guardian for all participants <18 years. Written assent was also obtained from all participants <18 years.

### 2.2 Participants

Target enrollment was 10 participants selected from among the patient population at the Comprehensive Sickle Cell Clinic at Children's Hospital of Michigan. Inclusion/exclusion criteria are listed in Table 1.

**TABLE 1:** SPICE (Sickle cell Pain: Intervention with Capsaicin Exposure) inclusion/exclusion criteria

Inclusion criteria	Ages 14–21 inclusive Sickle cell genotype of HbSS, HbSC, or HbSß^0^ Identifiable recurrent sites of pain where majority of prior acute pain episodes were localized Suggested NP component as evidenced by a symptom queried on painDETECT questionnaire
Exclusion criteria	Inclusion on a chronic transfusion program Major surgery prior 3 months Recurrent pain secondary to a non‐SCD condition Concurrent use of other topical analgesic medications Concurrent use of medications used in treating NP Treatment with hydroxyurea if no stable dose for the previous 3 months Pregnant females Known avascular necrosis at the site of pain to be assessed

### 2.3 Capsaicin

The 8% topical capsaicin, FDA approved for treatment of postherpetic neuralgia and diabetic peripheral NP, has been shown to be as efficacious as gabapentinoids in the treatment of NP with fewer side effects [24, 25]. A reversible excitotoxin, capsaicin achieves this effect after only a 1‐hour application period via agonistic inhibition of the TRPV1 receptor. It causes massive influx of Na^+^ and Ca^2+^ ions into the nerve terminal, triggering a strong action potential that consumes substance P stores to limit further sensitization at the DRG, and ultimately causing degeneration of targeted neuron axons. After a 3‐month period of analgesia, the axons regenerate fully with reduced (i.e., *not* upregulated) concentrations of TRPV1 [26, 27, 28, 29, 30, 31, 32, 33, 34]. The mechanism results in a hyperalgesia effect during and immediately after application, which is ameliorated effectively by the application of ice. In our study, the treated area was pretreated with 5% topical lidocaine as indicated in the package insert with ice packs offered on top of patches as needed.

### 2.4 Study activities

The study schema is shown in Supporting Information S1. At enrollment, participants identified their two most common sites of pain. No frequency threshold was required, as the treatment of chronic pain was not the primary aim, but rather the identification of localized sensitization even if only in the presence of recurrent acute pain. Seven visits were planned at 6‐week intervals. At each visit, participants completed the painDETECT questionnaire (Pfizer), a validated Likert scale‐based questionnaire validated for identification of NP based on patients’ reported pain experience. This was used as an exploratory endpoint assessing pain improvement. Mechanical QST was performed using an electronic von Frey instrument (Bioseb, Pinellas Park, FL) to determine localized pain threshold at the most common and second most common sites of pain. These sites remained the same after initial identification. Measurements using the instrument involve applying a gradually and consistently increasing force perpendicular to the skin with a hard plastic “Eppendorf” tip (0.5‐mm diameter contact point) mounted on the sensitive element. When the subject felt discomfort, he or she pressed a handheld button and the pressure in grams of force was recorded automatically. Mean pain threshold was obtained from three trials at each site. After testing during week 0, 12, and 24 visits, participants then underwent capsaicin application (Supporting Information S1).

### 2.5 Endpoints and analysis

The primary endpoint of safety was to be established by there being no intervention‐related serious adverse events, defined as grade 3 or greater according to the Common Terminology Criteria for Adverse Events. Feasibility/tolerability threshold was defined as completion of >80% of planned patch applications across all participants. The exploratory QST endpoint was assessed by the change in the ratio of the QST pain threshold at the most common (treated) painful site to the second most common (untreated) site as identified at enrollment, which we termed the Localized Peripheral Hypersensitivity Relief (LPHR) score. This value was chosen rather than the absolute change in the QST threshold at the treated site due to the potential variability in threshold from time point to time point related to various known triggers of pain in SCD: overall inflammation status, environment (temperature, wind speed), emotional state, and so forth. These would in theory affect both sites similarly. By measuring the threshold at a treated site relative to an untreated site, the participant functioned as his or her own control for the localized capsaicin intervention, thereby better isolating the contribution of localized TRPV1 differences to the pain phenotype. These data were analyzed using descriptive statistics with means and standard deviations, with paired samples *t*‐test used to determine significance of changes in pain threshold between enrollment and subsequent time points. Trends in painDETECT scores over time were obtained to explore their feasibility as trended data points to measure intervention effects.

## 3 RESULTS

Overall study population demographics and pain sites are shown in Table 2, with individual characteristics in Supporting Information S2. Nine participants completed visits 1–5 (weeks 0–24), and seven completed visit 6 (week 30) prior to study suspension due to COVID restrictions.

**TABLE 2:** Study population characteristics

Participant demographics summary	
Gender	
Male	5 (50%)
Female	5 (50%)
Age range (years)	14–19 (mean 16.6, median 16.5)
Race	
African American	9 (90%)
Hispanic	1 (10%)
Genotype	
HbSS	6 (60%)
HbSC	4 (40%)
Hydroxyurea status	
Yes	6
No	4
Most common painful site	
Lower extremity	7 (70%)
Back	3 (30%)

### 3.1 Safety and tolerability

There were no intervention‐related serious adverse events. There were 15 hospital admissions for vaso‐occlusive pain episodes (VOE) during the study period and one admission for delayed transfusion reaction. Only nine of the VOE admissions involved the treated site (See Supporting Information S2). All events were deemed unrelated to the intervention due to lack of temporal proximity to patch application and/or VOE occurring in an unrelated body segment. One participant requested that the patch be removed halfway through her second 60‐minute application (week 12) due to intolerance of burning sensation. Only mild erythema was noted at the site in terms of physical changes, and she had improvement upon patch removal. That participant did not have the third patch applied.

All other participants tolerated the patches well. They universally reported a hot sensation ranging from “warmth” to “burning.” Seven of 10 requested a cold pack during the first application with immediate relief. All participants noted residual heat hypersensitivity for 24–48 hours following removal, followed by return of normal sensation. During the week 12 visit, only one subject requested ice, and nine of 10 reported the heat sensation as being less intense than during the first application. Ninety‐three percent of planned study treatments were completed, with one mentioned above and another canceled due to COVID restrictions, indicating the patches were well tolerated overall.

### 3.2 Exploratory endpoints

#### 3.2.1 Participant report

At each visit, participants were asked to describe any changes in patterns of pain. Six of 10 felt after two treatments that the treated area was no longer their most common site of pain. Two participants were having mild vaso‐occlusive‐type pain at the time of their second patch application and stated the warm sensation “felt good.” One participant had significant pain on most days prior to enrollment. She had two separate prolonged admissions for VOE during the study period but stated during those admissions she had minimal pain in the treated area. This localized improvement in pain persisted for 9 months (last query) from the end of the study despite chronic pain at other locations.

#### 3.2.2 LPHR score

The above reports of pain improvement were supported by QST data. QST pain thresholds during the initial assessment ranged from 58.4 to 280.1 grams (g) of force at untreated sites and from 59.0 to 211.0 g at treated sites (see Supporting Information S3), and the LPHR score was <1.0 for eight of 10 participants. The other two scores were 1.01 and 1.02, respectively, indicating practically identical sensitivity at both sites. Overall LPHR range at week 0 was 0.67–1.02 (see Table 3).

**TABLE 3:** Individual Localized Peripheral Hypersensitivity Relief (LPHR) scores

**Participant (site 1/site 2)**	**Week 0 LPHR**	**Week 24 LPHR**	**LPHR change week 0 to week 24**
1 (Rt knee/Rt shin)	1.01	0.69	−0.32
2 (Rt ant thigh/Lt ant thigh)	0.89	0.90	+0.01
3 (Rt mid back/Lt mid back)	0.74	1.28	+0.50
4 (Rt ant thigh/Rt upper arm)	0.75	1.50	+0.75
5 (Rt shin/Lt shin)	1.02	1.08	+0.06
6 (Rt knee/Lt knee)	0.83	1.05	+0.22
7 (Rt low back/rt ant thigh)	0.67	1.23	+0.56
8 (Lt ant thigh/Rt upper arm)	0.86	NA	+0.20a
9 (Lt low back/Rt low back)	0.74	0.96	+0.22
10 (Lt knee/Rt knee)	0.87	1.22	+0.35
**Average**	**0.84**	**1.10**	**+0.26**

*Note*: The LPHR was <1.0 for 8/10 participants at enrollment, and only 3/10 participants at week 24, indicating most participants experienced reduced pain sensitivity at the treated site of pain relative to the untreated site. Note the measurements for participant 9 during week 18 were significant outliers relative to all other data points (identified by Dixon's *Q* test at a 95% confidence level) and were thus removed from the dataset prior to calculation of average scores. NA indicates visits that could not be completed due to COVID quarantine. Tested body sites for each participant listed in left column. For expanded data including raw QST measurements see Supporting Information S3.

^a^ Listed LPHR change for participant 8 is from week 0 to week 18.

**FIGURE 1:**
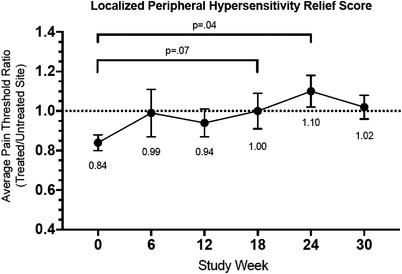
Localized Peripheral Hypersensitivity Relief (LPHR) score represents the difference in pain threshold by mechanical quantitative sensory testing (QST) at the most common site of vaso‐occlusive pain (QST_1_) as identified at time of enrollment relative to the second most common sites of vaso‐occlusive pain (QST_2_), calculated as QST_1_/QST_2 _= LPHR. Most common site of pain at enrollment was treated with high‐dose (8%) capsaicin after QST measurement at weeks 0, 12, and 24. Trend indicates an overall improvement in pain threshold at the treated area with an average improvement in LPHR from week 0 to week 18 of 0.16 (*p* = 0.07), and from week 0 to week 24 of 0.26 (*p* = 0.04). Of note, at time of study suspension for pandemic quarantine week 24 data were available for only nine of 10 participants and week 30 data for only seven of 10 participants

Among the nine participants who completed the week 24 (fifth) visit, eight of nine had improved scores at that time relative to the score at enrollment (Table 3). Of note, one participant had the week 24 visit canceled due to COVID but had two LPHR scores >1.0 at weeks 12 and 18 after having an enrollment score <1.0. The improvement in average LPHR score of 1.10 represents a 26% improvement in relative sensitivity (*p* = 0.04, comparing only the nine participants with week 24 scores), indicating that the pain threshold at the treated site was higher than that at the untreated site after two treatments (Figure 1). This is consistent with most participants stating the most common site of pain changed from study entry to completion.

The greatest improvement in sensitivity was a 75% improvement at the treated site relative to the untreated site for participant 4, and the smallest was 1% for participant 2. Only participants 1, 2, 5, and 9 experienced VOEs involving the treated sites, necessitating ED presentation or hospital admission (Supporting Information S2), and these same four had the poorest response as determined by LPHR score (Table 3). Hypersensitivity is known to be exacerbated during VOE, but the duration of that exacerbation has not been studied [35]. It is possible that pain sensitivity was exacerbated following these episodes, affecting subsequent testing, or either alternatively or additionally they had worse hypersensitivity at baseline via more complex mechanisms (possibly involving central sensitization) that contributes to more frequent/severe pain.

## 4 DISCUSSION

Establishing the safety of high‐dose capsaicin for use in SCD makes possible its consideration as both a probe of the pathophysiology of SCD‐related chronic pain and a potential treatment for the same. We have obtained pilot data and demonstrated a methodology to support its investigation for both uses.

### 4.1 Capsaicin as a mechanistic probe for SCD

While elements of the pathophysiology behind chronic pain development in SCD have been identified, the most important among these and the order in which they develop have not. TRPV1 is known to contribute to peripheral sensitization in mice with SCD, but this has not been previously demonstrated in humans, nor has the reason for TRPV1 upregulation been specifically defined. Studies of TRPV1 and NP in other disease states have demonstrated upregulation of this receptor's concentration and sensitivity on nociceptive neurons in response to repeated noxious stimuli [23, 36]. The baseline LPHR scores consistently being less than 1.0 in our study population suggest an association in distinct body sites between more frequent vaso‐occlusive pain and greater sensitization. Together with the reversal in relative sensitivity between the two sites following TRPV1 elimination, our results provide preliminary evidence that recurrent vaso‐occlusive pain episodes affect TRPV1 function similarly to previously studied noxious stimuli and that this effect occurs locally. This may contribute to the frequently seen “target sites” of pain in patients with SCD.

Multiple investigations of SCD patients have demonstrated alterations in the functional connectivity of brain networks known to be involved in pain processing and memory, and have suggested predictable patterns that arise in the presence of chronic pain [13, 14, 15, 37, 38]. This central sensitization represents one component of the complex pathophysiology of chronic pain in SCD, but is harder to test on a routine clinical basis and difficult to target therapeutically at the present level of understanding. Alternatively, TRPV1 upregulation is more specific and targetable [17]. Given the typical evolution of pain throughout the lifespan from recurrent acute pain to chronic pain and NP, it is reasonable to think that peripheral TRPV1 upregulation may represent an early stage in this pain evolution as opposed to central sensitization developing first and causing TRPV1 upregulation. Though this small pilot study only demonstrates that TRPV1 contributes to pain and the removal of this factor reduces localized mechanical sensitivity, we have demonstrated an ability to isolate its effect on pain phenotypes in patients with SCD, which will be beneficial for future translational investigations of pain mechanisms. Our method, which we now know to be safe and well tolerated, provides a means of interrogating the pain pathway by “turning off” a targetable component and testing its effect on other components.

### 4.2 Therapeutic potential of capsaicin in SCD

As the model of pain in SCD grows more complex, so must the management approach. The rate at which patients receive medications targeting NP in particular as a cause of chronic pain lags far behind its likely prevalence among patients with SCD [4, 5, 39]. The recent guidelines from the American Society of Hematology for the treatment of SCD‐associated chronic pain include the use of several medications recommended for NP treatment in the broader pain literature, but the recommendations are based almost entirely on data from other disease processes due to the lack of clinical trials targeting NP in SCD [40]. Capsaicin was not included in those guidelines for similar reasons.

The previously identified peripheral processes that contribute to NP in SCD combined with our data regarding pain frequency‐related localized sensitivity make it a compelling candidate for further therapeutic investigation. Our pilot data provide early subjective and objective evidence suggesting high‐dose (8%) topical capsaicin is safe and well‐tolerated and may be efficacious in reducing pain for patients with SCD regardless of true positive screening for NP on painDETECT. There was no correlation between participants’ score on the painDETECT questionnaire at enrollment and their subsequent improvement in LPHR scores or subjective improvement in pain, and in fact the two participants (1 and 2) with the highest painDETECT scores at baseline had the least improvement in peripheral sensitivity by LPHR over time. Our hypothesized reason for this is two‐fold. First, perhaps SCD patients with nervous system malfunction sufficient to cause screen‐positive NP have central sensitization beyond what can be affected by reduction in TRPV1 expression. Second, it is likely that a component of central and/or peripheral sensitization exists in more patients with SCD than just those with positive screens for NP.

The recently proposed concept of “nociplastic pain” provides a useful framework to think about more subtle changes in pain sensitivity and chronic SCD pain overall that may not be detected on screening tools [41, 42]. Nociplastic pain is defined as “pain that arises from altered nociception, despite no clear evidence of actual or threatened tissue damage causing the activation of peripheral nociceptors or evidence for disease or lesion of the somatosensory system causing the pain” [41]. Peripheral sensitization at least partially mediated by TRPV1 upregulation would fall under this category [19, 22, 43]. Our pilot data, which identified localized baseline hypersensitivity related to more frequent localized vaso‐occlusive pain, suggest nociplastic pain may be an important intermediate step in the development of chronic SCD pain that is worthy of further investigation. If proven to be a significant factor in chronic pain development, there may be more patients than just those screening positive for NP based on established PROs who would benefit from interventions targeting a damaged or malfunctioning nervous system. The integration of novel assessment tools like the LPHR score with PROs specifically designed for SCD, once further validated, will be valuable in refining pain interrogation and treatment algorithms for chronic nociceptive, nociplastic, and neuropathic SCD pain.

## CONFLICT OF INTEREST

Alexander K. Glaros receives honoraria from Global Blood Therapeutics. Michael U. Callaghan receives honoraria from Roche/Genentech, Takeda, Sanofi, Pfizer, Bayer, Global Blood Therapeutics, Bluebird Bio, Uniqure, Spark, Biomarin, Kedrion, Grifols, and Hema Biologics; he serves on the speaker bureau for Roche/Genentech, Takeda, Bayer, Global Blood Therapeutics, Biomarin, and NovoNordisk; and receives research funding from Pfizer. Ahmar U. Zaidi receives honoraria from Global Blood Therapeutics, Novartis, Emmaus Life Sciences, Cyclerion, bluebird bio, Chiesi, NovoNordisk, and Agios; and serves on the speaker bureau for Global Blood Therapeutics and receives research funding from Emmaus Life Sciences and Novartis.

## AUTHORSHIP CONTRIBUTIONS

All authors contributed to the design of the study. Alexander K. Glaros, Michael U. Callaghan, and Ahmar U. Zaidi contributed to the recruitment of patients and their management. Alexander K. Glaros performed the statistical analysis with input and review by all authors. Alexander K. Glaros drafted the manuscript and all authors read, revised, and approved it prior to submission.

## PATIENT CONSENT STATEMENT

Written informed consent was obtained from all participants ≥18 years old and from a legal guardian for all participants <18 years. Written assent was also obtained from all participants <18 years.

## ETHICS STATEMENT

This study was approved by the Institutional Review Boards of Wayne State University and the Detroit Medical Center as an open‐label, single‐arm safety investigation of off‐label use of an FDA‐approved medication.

## Supporting information

Supporting Information S1

Supporting Information S2

Supporting Information S3

## Data Availability

The data that support the findings of this study are available from the corresponding author upon reasonable request.
