# Supplementary material for: Targeting TRPV1 activity via high‐dose capsaicin in patients with sickle cell disease
Source: EJHaem. 2022 Jul 19;3(3):653–9. doi: 10.1002/jha2.528 (PMC9421981; doi:10.1002/jha2.528)
Supplement: Supplementary file 1 — Supporting Information S1 [file JHA2-3-653-s002.docx]

**Supplement 1: Methodology**


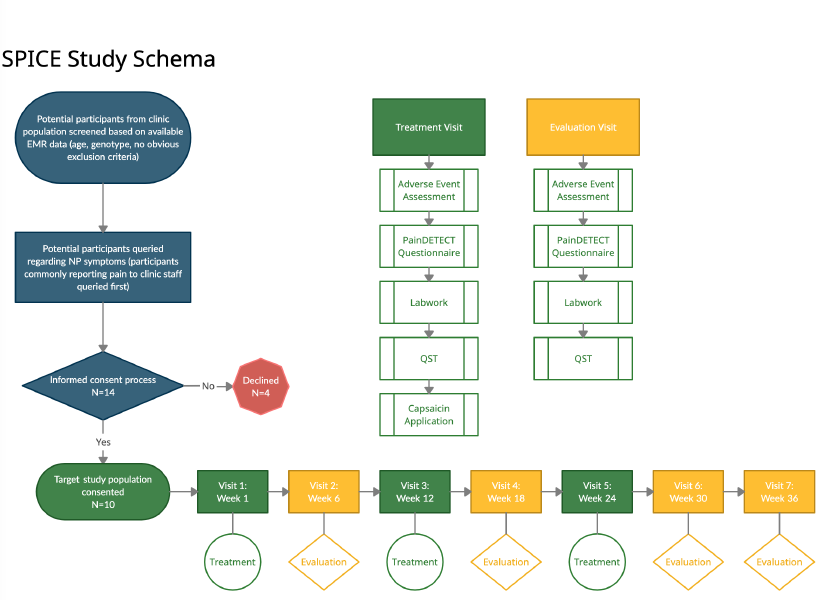


**Procedures**

**Capsaicin Application:**

Participants identified their most frequent site of pain at time of enrollment. This was established as the site to be treated for the duration of the study. During treatment visits, after all assessments were completed, two 5% topical lidocaine patches were applied side by side over the site to be treated. Two patches were used to cover the full span covered by the larger capsaicin patch. Lidocaine patches were left in place for 30 minutes. The area was traced with a marker prior to lidocaine patch removal and cleansing of the area with soap and water. The capsaicin patch was trimmed as necessary to fit within the traced area. Capsaicin patch was then applied and covered with an ace wrap or medical tape to ensure adherence and to prevent contamination of participant’s hands. The patch was left in place for 60 minutes before being removed. The area was then cleansed with the cleansing gel provided in the capsaicin packaging. During the 60-minute application period, if participant requested it due to an uncomfortable heat sensation, an ice pack was provided and applied on top of the covered patch.
