# Supplementary material for: Targeting TRPV1 activity via high‐dose capsaicin in patients with sickle cell disease
Source: EJHaem. 2022 Jul 19;3(3):653–9. doi: 10.1002/jha2.528 (PMC9421981; doi:10.1002/jha2.528)
Supplement: Supplementary file 2 — Supporting Information S2 [file JHA2-3-653-s003.docx]

**Supplement 2: Individual Participant Information**

| **Participant** | **Genotype** | **Age** | **Gender** | **Daily HU Dose** | **Treated Pain Site** | **Untreated Pain Site** | **VOCs at treated site (9 months prior to enrollment)** | **VOCs at treated site (during study period)** |
| --- | --- | --- | --- | --- | --- | --- | --- | --- |
| **1** | SC | 18 | F | 25mg/kg | Right knee | Right shin | 3 | 2 |
| **2** | SC | 17 | F | NA | Right anterior thigh | Left anterior thigh | 1 | 1 |
| **3** | SS | 16 | M | 33mg/kg | Right mid back | Left mid back | 0 | 0 |
| **4** | SS | 19 | F | 15mg/kg | Right anterior thigh | Right upper arm | 0 | 0 |
| **5** | SC | 19 | M | 25mg/kg | Right shin | Left shin | 7 | 5 |
| **6** | SS | 14 | F | 26mg/kg | Right knee | Left knee | 0 | 0 |
| **7** | SC | 15 | M | NA | Right low back | Right anterior thigh | 0 | 0 |
| **8** | SS | 18 | M | NA | Left anterior thigh | Right upper arm | 1 | 0 |
| **9** | SS | 15 | F | NA | Left low back | Right low back | 4 | 1 |
| **10** | SS | 15 | M | 22mg/kg | Left knee | Right knee | 0 | 0 |
