# Supplementary material for: Targeting TRPV1 activity via high‐dose capsaicin in patients with sickle cell disease
Source: EJHaem. 2022 Jul 19;3(3):653–9. doi: 10.1002/jha2.528 (PMC9421981; doi:10.1002/jha2.528)
Supplement: Supplementary file 3 — Supporting Information S3 [file JHA2-3-653-s001.docx]

| **Participant** (Site 1/Site 2) | **Week 0 LPHR**  (QST_1_/QST_2_) | **Week 6 LPHR**  (QST_1_/QST_2_) | **Week 12 LPHR**  (QST_1_/QST_2_) | **Week 18 LPHR**  (QST_1_/QST_2_) | **Week 24 LPHR**  (QST_1_/QST_2_) | **Week 30 LPHR**  (QST_1_/QST_2_) | **LPHR Change**  **Week 0 to Week 24** |
| --- | --- | --- | --- | --- | --- | --- | --- |
| **1** (Rt knee/Rt shin) | **1.01** (59.0/58.4) | **1.45** (139.4/96.4) | **0.46** (45.8/98.6) | **0.80** (73.2/91.5) | **0.69** (56.6/81.8) | **1.08** (70.5/65.4) | **-0.32** |
| **2** (Rt ant thigh/Lt ant thigh) | **0.89** (140.5/157.5) | **0.69** (73.1/105.7) | **1.03** (102.4/99.6) | **1.20** (99.1/82.5) | **0.90** (80.9/90.1) | **0.89** (86.9/98.0) | **+0.01** |
| **3** (Rt mid back/Lt mid back) | **0.74** (78.0/105.4) | **1.77** (92.9/52.6) | **0.71** (62.3/88.1) | **1.03** (51.5/50.0) | **1.28** (80.6/62.9) | **1.24** (81.6/66.0) | **+0.50** |
| **4** (Rt ant thigh/Rt upper arm) | **0.75** (211.0/280.1) | **1.09** (188.9/172.9) | **1.26** (227.3/180.0) | **0.97** (163.3/168.3) | **1.50** (205.7/137.3) | **1.03** (180.0/174.1) | **+0.75** |
| **5** (Rt shin/Lt shin) | **1.02** (86.5/85.1) | **0.70** (85.4/121.3) | **0.96** (106.6/111.1) | **0.85** (79.4/93.0) | **1.08** (102.1/94.2) | **0.80** (102.3/128.2) | **+0.06** |
| **6** (Rt knee/Lt knee) | **0.83** (132.1/160.1) | **0.97** (122.3/126.5) | **1.10** (106.8/97.0) | **1.26** (138.0/109.8) | **1.05** (103.8/99.0) | **0.93** (116.6/125.0) | **+0.22** |
| **7** (Rt low back/rt ant thigh) | **0.67** (111.6/166.3) | **0.46** (67.2/146.7) | **0.83** (115.3/138.9) | **0.69** (92.7/135.2) | **1.23** (105.8/86.0) | **1.16** (104.2/90.1) | **+0.56** |
| **8** (Lt ant thigh/Rt upper arm) | **0.86** (189.9/221.3) | **0.85** (133.7/157.2) | **1.03** (167.7/163.0) | **1.06** (203.3/191.0) | **NA** | **NA** | **+0.20**** |
| **9** (Lt low back/Rt low back) | **0.74** (197.4/267.6) | **0.96** (177.8/185.0) | **1.00** (226.9/227.2) | **0.62*** (319.0/518.3) | **0.96** (144.8/151.2) | **NA** | **+0.22** |
| **10** (Lt knee/Rt knee) | **0.87** (155.2/178.8) | **0.99** (105.5/106.8) | **0.98** (110.9/113.3) | **1.49** (166.4/111.6) | **1.22** (145.2/119.4) | **NA** | **+0.35** |
| **Average** | **0.84** | **0.99** | **0.94** | **1.00** | **1.10** | **1.02** | **+0.26** |

***Supplement 3. Individual Localized Peripheral Hypersensitivity Relief (LPHR) Scores. LPHR is a value representing the ratio between the pain threshold at two sites as measured by quantitative sensory testing. LPHR scores in this table represent the difference in pain threshold between a commonly painful site treated with capsaicin and one not treated with capsaicin over time. The LPHR was <1.0 for 8/10 participants at enrollment, and only 3/10 participants at week 24, indicating most participants experienced improvement in pain threshold at the treated site of pain relative to the untreated site. *Note the measurements for participant 9 during week 18 were significant outliers relative to all other datapoints (Treated QST threshold =319g, Untreated QST threshold= 518.3) identified by Dixon’s Q Test at a 95% confidence level, and were thus removed from the dataset prior to calculation of average scores. ** Listed LPHR change for participant 8 is from week 0 to week 18. NA indicates visits that could not be completed due to COVID quarantine. Tested body sites for each participant listed in left column. QST_1_- value at site 1 (most painful, treated); QST_2_- value at site 2 (2^nd^ most painful, untreated) site.***
